# Self-reported colorectal cancer screening of Medicare beneficiaries in family medicine vs. internal medicine practices in the United States: a cross-sectional study

**DOI:** 10.1186/1471-230X-12-23

**Published:** 2012-03-21

**Authors:** Angela Y Higgins, Anna R B Doubeni, Karon L Phillips, Adeyinka O Laiyemo, Becky Briesacher, Jennifer Tjia, Chyke A Doubeni

**Affiliations:** 1 Department of Family Medicine and Community Health, University of Massachusetts (UMass) Medical School, 55 Lake Avenue North, Worcester, MA 01655, USA; 2 Program on Aging and Care, Scott & White Memorial Hospital, 2401 South 31st. Street, Temple, TX 76508, USA; 3 Division of Gastroenterology, Department of Medicine, Howard University College of Medicine, 2041 Georgia Avenue, NW, Washington, DC 20060, USA; 4 Meyers Primary Care Institute and Division of Geriatrics, UMass Medical School, 377 Plantation Street, Worcester, MA 01605, USA

**Keywords:** Colorectal cancer screening, Primary care physicians, Colonoscopy, Fecal occult blood test

## Abstract

**Background:**

The benefit of screening for decreasing the risk of death from colorectal cancer (CRC) has been shown, yet many patients in primary care are still not undergoing screening according to guidelines. There are known variations in delivery of preventive health care services among primary care physicians. This study compared self-reported CRC screening rates and patient awareness of the need for CRC screening of patients receiving care from family medicine (FPs) vs. internal medicine (internists) physicians.

**Methods:**

Nationally representative sample of non-institutionalized beneficiaries who received medical care from FPs or internists in 2006 (using Medicare Current Beneficiary Survey). The main outcome was the percentage of patients screened in 2007. We also examined the percentage of patients offered screening.

**Results:**

Patients of FPs, compared to those of internists, were less likely to have received an FOBT kit or undergone home FOBT, even after accounting for patients' characteristics. Compared to internists, FPs' patients were more likely to have heard of colonoscopy, but were less likely to receive a screening colonoscopy recommendation (18% vs. 27%), or undergo a colonoscopy (43% vs. 46%, adjusted odds ratios [AOR], 95% confidence interval [CI]-- 0.65, 0.51-0.81) or any CRC screening (52% vs. 60%, AOR, CI--0.80, 0.68-0.94). Among subgroups examined, higher income beneficiaries receiving care from internists had the highest screening rate (68%), while disabled beneficiaries receiving care from FPs had the lowest screening rate (34%).

**Conclusion:**

Patients cared for by FPs had a lower rate of screening compared to those cared for by internists, despite equal or higher levels of awareness; a difference that remained statistically significant after accounting for socioeconomic status and access to healthcare. Both groups of patients remained below the national goal of 70 percent.

## Background

Screening has been shown to decrease the risk of mortality for colorectal cancer (CRC) [1-4]. Although the use of CRC screening has increased in the US, particularly over the past decade [5,6], for many groups, screening rates are below the Healthy People goal of 70% [7]. Primary care physicians (PCPs) play an important role in the delivery of CRC screening services [6,8-12] by advising, recommending, performing and/or referring patients for screening [13]. It is therefore not surprising that studies have consistently reported a strong association between healthcare provider recommendations for and receipt of CRC screening for persons with a usual source of care [8,9,14]. This supports the hypothesis that variations in the delivery of CRC screening services among PCPs have a negative impact on efforts to increase screening rates in the US. However, there are very few published studies on PCP-related variations in CRC screening that simultaneously assess screening rates and physicians' delivery of screening, as reported by patients.

Despite the limited literature on PCP-related variation in screening, the existing evidence about differing quality of preventive health care among PCPs [15-18] suggests that similar differences in quality of CRC screening may contribute to underuse of screening in some populations. One study, which included analyses on CRC testing by PCP specialty, found that patients receiving their usual routine care from family/general practitioners were less likely to have had CRC testing than those receiving care from a general internists [18]. However, the findings of that study were limited by the use of insurance claims data alone and the exclusion of fecal occult blood test (FOBT) use in the definition of CRC screening. Thus, there is paucity of published studies that simultaneously addressed both a patient's awareness and use of CRC screening services and a physician's recommendation and delivery of CRC screening through interviews with patients. This would lead to a better understanding if it is clarified whether or not low rates of screening are the result of patient factors such as education or income or a physician's failure to offer screening. Additionally, understanding the variations in screening practices according to PCP specialties will provide insights into some of the reasons behind suboptimal rates of CRC screening in the US. A key question is whether a particular PCP specialty group has achieved the US national CRC screening objectives.

In this study, we compared the CRC screening practices of patients receiving care from FPs to those receiving care from internists. We examined individual's awareness of CRC screening tests and the utilization of such screening, according to whether the patient received their usual medical care from an FP or internist, and related those differences to patient factors.

## Methods

The data for this study were obtained from the Medicare Current Beneficiary Survey (MCBS) and matched Medicare claims. The design and data collection methods for the MCBS are described in detail previously [19,20]. In brief, the MCBS is an ongoing annual survey with four-year rotating cohorts of nationally representative samples of Medicare beneficiaries who are interviewed in-person three times a year [19]. We used data on non-institutionalized beneficiaries who were 50-75 years of age at the beginning of 2006, were continuously enrolled in Medicare, participated in the interviews in both 2006 and 2007, and had no personal history of renal disease or CRC in 2006 or 2007. Our study population therefore also included persons 50-64 years of age who were in Medicare as a result of disability. Our upper age cutoff was based on current U.S. Preventive Services Task Force CRC screening guidelines [21,22]. This study was reviewed by the institutional review board (IRB) of the University of Massachusetts Medical School (Worcester, MA) and was considered exempt from a full IRB review.

### Data elements

Each survey year, the MCBS collects data on participants including age, gender and marital status; residence in metropolitan service areas (MSA); highest level of educational achievement (less than high school vs. others); household income (<$25,000 vs. others); primary language (English vs. all other languages); the type of health insurance coverage (supplemental vs. no supplemental insurance); and delaying medical care due to cost [19,20]. These factors are known to be associated with use of CRC screening [23]. Additional data collected includes beneficiary employment, history of non-skin cancers and self-rated general health status (which was used as proxy for wellness to undergo screening).

### Specialty of the usual care primary care physician

The primary predictor in our analyses was the specialty of PCP that a beneficiary usually sees for medical care (FP vs. internist). During the fall of each year, the MCBS collects information about the "particular medical person or ... clinic [a beneficiary] usually goes to when [he/she is]... sick or [for] advice about ... health", and the specialty of the particular doctor he/she usually sees. Among the potentially eligible subjects for this study, 1,922 patients reported their usual physician was either an FP or internist. We then used the Unique Physician Identification Numbers from Medicare claims to match 1,354 other patients to an FP or internist. When both specialties were identified for a particular subject (n = 208), the PCP with the greater number of services rendered was assigned: no ties were observed [24].

### Measures of colorectal cancer awareness and screening

**Figure 1:**
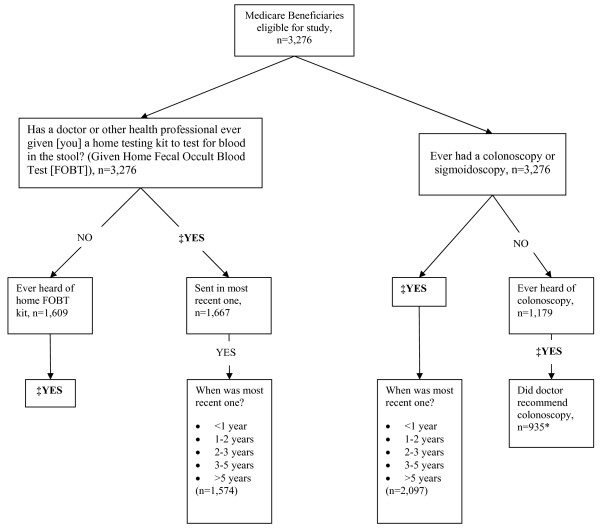
**Design of CRC testing questions on the 2007 Medicare Current Beneficiary Survey with analytic samples (n = 3,276)**. **‡**Participants who had previously heard of or received a screening by FOBT (fecal occult blood test) or sigmoidoscopy/colonoscopy were asked if they knew that Medicare helps to pay for colon cancer screening, n = 3,130. *Those who have never heard of colonoscopy were considered not to have previously received a recommendation and thus included in the dominator for this analysis.

In the fall of 2007, MCBS respondents 50 years of age or older, who did not report a history of CRC, were asked whether they had ever had CRC screening test (sigmoidoscopy, colonoscopy, and/or a home FOBT), and if so, the date of the most recent test (as shown in Figure 1.) We defined a nominal screening variable by first considering the receipt of a sigmoidoscopy/colonoscopy within five years of the interview date and then FOBT within one year, in a mutually exclusive manner. We also created a combined outcome of CRC screening defined as receiving colonoscopy or sigmoidoscopy within 5 years and/or FOBT within one year. This approach was based on how questions were asked on the MCBS as described previously [20]. Respondents were also asked if they were ever given an FOBT kit (categorized as yes vs. no), and those who had received an FOBT kit were then asked if they had returned the last kit (categorized as yes vs. no). Patients who had not previously received a kit were asked if they had "ever heard of this home testing kit" (categorized as yes vs. no). In addition to questions on FOBT use, those who had not previously undergone a colonoscopy or sigmoidoscopy were asked if they had "ever heard of" sigmoidoscopy/colonoscopy and if so, whether his/her physician recommended that he/she should have the exam (categorized as yes vs. no). Participants who had previously heard of or received CRC screening were asked: "Before today, did you know that Medicare now helps pay for the cost of screening tests".

### Data analyses

Two-by-two contingency tables and the Wald chi-square test were used to compare beneficiaries' characteristics, awareness and knowledge of CRC screening according to PCP specialty (FP vs. internist). Specifically, we examined differences in awareness of CRC, colonoscopy/sigmoidoscopy, home FOBT kit, or Medicare's coverage for CRC screening. Analyses on knowledge of Medicare's coverage for CRC screening were stratified according to prior history of colonoscopy/sigmoidoscopy. We also examined for differences in whether or not a beneficiary returned his/her last home FOBT kit.

Multinomial regression models were used to determine whether use of an FOBT alone within one year, or colonoscopy/sigmoidoscopy alone within five years differed according to PCP specialty. We then used logistic regression models to examine the association between PCP specialty and the combined outcome of any CRC screening exam. Further, we examined two additional outcomes that involve direct healthcare provider participation as reported by patients: whether an eligible beneficiary was given an FOBT kit, or received a colonoscopy recommendation. Several subgroup and sensitivity analyses were also performed.

Individual enrollees were the unit of analyses, and we used data on beneficiaries' usual PCP and covariates from the 2006 survey for the analyses. The covariates included in the regression models (as shown in Table 1) were based on previous studies [20]. We used cross-sectional survey weights in all analyses and variance estimation accounted for the complex survey design. The analyses were performed using STATA version 12.

**Table 1:** Characteristics of the study population by primary care physician specialty, MCBS 2006-2007 (n = 3,276)

Characteristic, %	General Internist, n = 1,624	Family Physicians, n = 1,652	Wald test p- values
**Age, years**			

*50-54*	5.1	5.1	
	
*55-64*	13.2	15.6	0.24
	
*65-69*	40.0	40.6	
	
*70-75*	41.7	38.7	

**Race/Ethnicity**			

*Whites*	77.8	83.0	
	
*Blacks*	10.5	8.4	0.02
	
*Hispanics*	7.8	5.7	
	
*Others*	3.9	3.0	

Female	55.5	54.3	0.54

Married or living together	63.3	63.4	0.94

Residing in Metropolitan Service Area	80.6	66.2	<0.01

Had less than high school diploma	19.1	26.6	<0.01

Annual household income <$25,000	44.4	49.1	0.03

Language of interview, English	95.1	98.2	<0.01

Working at a job	17.8	17.0	0.58

Delayed medical care due to cost	10.8	12.7	0.12

Had supplemental health insurance	74.8	69.7	0.01

Had a history of non-skin cancer	15.4	13.3	0.10

General health fair-to-poor	27.1	28.0	0.54

There were a total of 527 beneficiaries in the sample who were in Medicare because of disabilities. Compared to persons in Medicare because of age-eligibility, a higher proportion of those with disability were non-Hispanic blacks (8% vs. 18%) or were Hispanic (0.8% vs.2%) (*p*-value <0.01). They were less likely to be married (*p*-value <0.01), have supplemental insurance (*p*-value <0.01) or have received a high school diploma (*p*-value <0.01). Beneficiaries with disability were more likely to report fair or poor health (*p*-value <0.01), or have an annual income of < $25,000 (*p*-value <0.01). The proportion receiving care from FPs (53%) or internists (47%) was similar irrespective of disability status.

## Results

### Characteristics of the study population

A total of 3,276 subjects, representing a weighted sample of about 14.3 million Medicare enrollees, were included in the analyses. Of these subjects, 2,629 (81%) were non-Hispanic whites, 320 (9%) were non-Hispanic blacks, 224 (7%) were of Hispanic ethnicity and 103 (3%) were of other or unknown race/ethnicity.

After applying sampling weights, about 49% of beneficiaries received their usual care from FPs and 51% from internists. The characteristics of the study population according to PCP specialty are shown in Table 1. Compared to internists, a lower percentage of FPs' patients were blacks or Hispanics, or resided in a Metropolitan Service Area (MSA). A lower percentage of patients receiving care from FPs had high school diploma or higher, >$25,000 in annual household income, or supplemental insurance. FPs' patients were more likely to have been interviewed in English.

### Awareness and receipt of home FOBT kits

**Figure 2:**
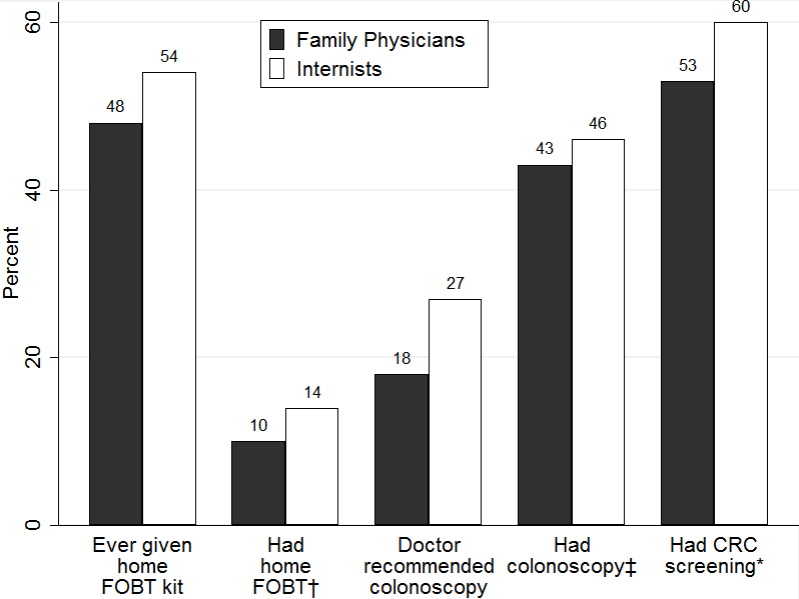
**Percentages of colorectal cancer screening outcomes by usual primary care physician specialty, MCBS 2006-2007 (n = 3,276)**. **Notes: **†FOBT = Fecal occult blood test. ‡Refers to colonoscopy or sigmoidoscopy. *Screening defined as receipt of colonoscopy or sigmoidoscopy within 5 years and/or FOBT within 1 year. All *p*-values <0.01

Among beneficiaries who had not previously had a home FOBT, about one-half had heard of the test, which was similar for patients of FPs and internists (*p*-value = 0.15, Table 2). However, a lower percent of patients receiving care from FPs (48%) than from internists (54%) had previously been given an FOBT kit (Figure 2). This difference was statistically significant even after adjusting for other covariates (adjusted odds ratio [AOR], 95% confidence intervals [CI]--0.82, 0.69-0.96). Of those who had previously received an FOBT kit, about 95% returned their most recent one for testing and again, this was similar for patients of both FPs and internists (*p*-value = 0.27, Table 2).

**Table 2:** Knowledge and awareness of colorectal cancer screening by primary care physician specialty, MCBS 2006-2007 (n = 3,276)

Interview items	CRC screening knowledge and awareness by Specialty % (95% CI)		
	
	General Internist, n = 1,624	Family Physicians, n = 1,652	Wald test p-values
Previously heard of colon cancer	86.6 (84.2-89.0)	86.4 (82.8-90.0)	0.91

Previously heard of colonoscopy*	76.6 (72.3-80.9)	82.1 (78.8-85.4)	0.04

Previously heard of home FOBT kit	50.1 (44.8-55.3)	54.4 (50.4-58.3)	0.15

Returned most recent FOBT kit	94.9 (93.2-96.7)	93.5 (91.5-95.5)	0.27

Knew of Medicare's benefit for screening stratified by prior receipt of colonoscopy*			

*Previously had colonoscopy*	57.9 (54.0-61.8)	54.1 (49.9-58.2)	0.12

*Not previously had colonoscopy*	34.4 (29.7-39.1)	31.7 (28.1-35.3)	0.34

*Refers to colonoscopy or sigmoidoscopy; *FOBT *Home fecal occult blood test

### Awareness of colonoscopy/sigmoidoscopy and receipt of recommendation

Among patients who had not previously undergone colonoscopy/sigmoidoscopy, a higher percentage of patients receiving care from FPs than from internists had heard of colonoscopy or sigmoidoscopy (82% vs. 77%, *p*-value = 0.04, Table 2). Only 18% of patients receiving care from FPs had received a colonoscopy recommendation compared to 27% receiving care from internists (Figure 2). The difference in receipt of colonoscopy recommendation remained statistically significant even after accounting for the effects of other covariates (AOR, CI--0.59, 0.44-0.88, Table 3). Compared to persons in Medicare because of age-eligibility, disabled beneficiaries were less likely to report having received a recommendation for colonoscopy/sigmoidoscopy (25% vs. 13%, *p *< .01).

**Table 3:** Unadjusted and adjusted estimates of the association between PCP specialty and CRC screening practices, MCBS 2006-2007 (n = 3,276)

Screening outcomes by PCP specialty	Unadjusted percent (95% CI)	Odds ratios (95% CI)	
		
		Unadjusted	Adjusted
*Logistic regression models analyses*			

**Given home FOBT kit**			

Internists	54.4 (51.2-57.8)	1.00 (ref)	1.00 (ref)

Family physicians	47.5 (44.2-50.9)	0.76 (0.65,0.89)	0.82 (0.69,0.96)

**Recommendation for colonoscopy/sigmoidoscopy**			

Internists	27.1 (22.6-31.5)	1.00 (ref)	1.00 (ref)

Family physicians	18.0 (14.9-21.2)	0.59 (0.44,0.80)	0.64 (0.47,0.86)

*Multinomial models analyses with unscreened as common reference group*			

**Had home FOBT within a year**			

Internists	14.0 (12-15.9)	1.00 (ref)	1.00 (ref)

Family physicians	9.8 (8.4-11.2)	0.59 (0.47-0.74)	0.65 (0.51-0.81)

**Had colonoscopy within 5 years**			

Internists	46.3 (43.4-49.2)	1.00 (ref)	1.00 (ref)

Family physicians	42.7 (39.7-45.8)	0.77 (0.65-0.92)	0.84 (0.71-1.00)

*Logistic regression models analyses*			

**Had CRC screening**			

Internists	60.2 (57.5-63.0)	1.00 (ref)	1.00 (ref)

Family physicians	52.5 (49.6-55.4)	0.73(0.62,0.86)	0.80 (0.68,0.94)

* Adjusted for age, race, ethnicity, sex, marital status, language of the interview, residence in metropolitan service area, education, annual household income, delayed medical care due to cost, supplemental insurance, work status, history of non-skin cancer and general health status

### Association between PCP specialty and use of CRC screening

The percentage of patients having FOBT within the past year was lower for those receiving care from FPs (10%) as compared to patients of internists (14%) (Figure 2 and Table 3). Similarly, the percentage of patients who had undergone a colonoscopy/sigmoidoscopy within the past 5 years was slightly lower for patients of FPs (43%) than it was for patients of internists (46%). Analyses on the combined screening outcome confirmed that a lower percentage of beneficiaries receiving care from FPs (52%) had undergone CRC screening, compared to patients of internists (60%) (Figure 2). The PCP specialty differences in receipt of an FOBT alone (AOR, CI-0.65, 0.51-0.8), colonoscopy/sigmoidoscopy alone (AOR, CI-0.84, 0.71-1.00), or any screening exam, (AOR, CI-0.80, 0.68-0.94) remained statistically significant even after controlling for patients' characteristics including education, income, health insurance coverage and place of residence (Table 3).

In subgroup analyses, the highest rates of screening were observed among beneficiaries with annual household incomes of $25,000 or higher receiving care from internists (68%). The lowest rates were among persons 50-54 years of age (34%), those on disability (46%) or those without a high school diploma (41%) who had received care from FPs (Table 4). In the subgroups of patients stratified by race and ethnicity, residence in MSA, income level, educational achievement and health insurance type, patients receiving care from FPs had consistently lower odds of undergoing screening. The largest relative differences were observed among enrollees aged 50-64 years (OR, CI −0.50, 0.27-0.89), blacks (OR, CI--0.62, 0.41-0.92) or people with supplemental insurance (OR, CI--0.77, 0.68-0.87).

**Table 4:** Stratified adjusted estimates of the association between PCP specialty and use of CRC screening, MCBS 2006-2007 (n = 3,276)

Characteristics	Percent screened (95% CI]		Odds ratios for FPs relative to internists (95% CI)
	
	Internists	Family Physician	
**Age, yrs**			

50-54	49.7 (41.5-57.9)	33.6 (23.5-43.7)	**0.50 (0.27-0.89)**

55-64	48.6 (42.5-54.7)	45.6 (39.7-51.4)	0.93 (0.65-1.33)

65-69	62.2 (59.8-64.7)	56.4 (53.4-59.4)	0.87 (0.75-1.01)

70-75	62.8 (60.0-65.5)	54.0 (51.1-56.9)	0.76 (0.64-0.90)

**Race/Ethnicity**			

Whites	62.0 (59.9-64.1)	54.5 (52.2-56.8)	0.83 (0.73-0.94)

Blacks	60.3 (55.1-65.5)	44.1 (35.3-52.8)	**0.62 (0.41-0.92)**

Hispanics	50.4 (44.1-56.6)	43.1 (35.3-51.0)	0.75 (0.47-1.19)

Others	41.6 (31.9-51.4)	41.1 (28.5-53.6)	1.08 (0.58-2.01)

**Residing in Metropolitan Service Area**			

No	53.9 (50.1-57.8)	46.0 (41.9-50.0)	0.80 (0.63-1.01)

Yes	61.6 (59.6-63.6)	55.9 (53.7-58.1)	0.81 (0.72-0.91)

**Less than high school diploma**			

No	62.8 (60.7-64.8)	56.8 (54.8-58.9)	0.81 (0.72-0.91)

Yes	48.8 (44.8-52.8)	40.9 (37.2-44.6)	0.79 (0.63-0.98)

**Annual household income**			

>$25,000	67.6 (65.6-69.5)	61.2 (58.8-63.7)	0.80 (0.70-0.92)

<$25,000	50.8 (47.7-54.0)	43.7 (40.4-47.1)	0.81 (0.68-0.97)

**Had supplemental health insurance**			

No	47.5 (43.6-51.3)	43.7 (39.4-48.0)	0.91 (0.73-1.14)

Yes	64.4 (62.4-66.3)	56.4 (54.3-58.5)	**0.77 (0.68-0.87)**

### Sensitivity analyses

Multivariable analyses, restricted to those who had reported on the MCBS that an FP (n = 935) or internist (n = 987) was their usual physician, found that enrollees seeing FPs were 0.80 (CI: 0.69-0.92) times as likely as those seeing internists to undergo screening. Analyses were also performed with CRC screening defined as receipt of sigmoidoscopy/colonoscopy within 2 years and/or FOBT within 1 year, while excluding persons who had sigmoidoscopy/colonoscopy more than 2 years previously (n = 1,007). Compared to internists, FPs' patients were 0.79 (CI: 0.66-0.96) times as likely to have been screened. Analyses restricted to enrollees 65-75 years old did not change the findings.

## Discussion

This study examined the delivery and receipt of CRC screening among a nationally representative sample of Medicare beneficiaries according to the specialty of their usual PCP. We found that the use of CRC screening tests, defined in our study as having completed FOBT testing within one year or sigmoidoscopy or colonoscopy within 5 years, was lower than the national goal of 70% for both internists and family physicians despite high levels of CRC screening awareness among beneficiaries. We found that patients receiving care from FPs had a lower rate of testing than those receiving care from internists, despite equal or higher awareness of screening tests among FPs' patients. PCP specialty differences were observed for all testing outcomes examined and remained statistically significant even after accounting for socioeconomic status of enrollees and factors related to access to health care. Surprisingly, the magnitude of the differences in use of screening tests was similar across many of the subgroups examined. The stability of the results across multiple outcomes, subgroups and multiple sources of information strengthens our findings.

We found a wide spectrum in terms of testing rates in this population. In 2007, 68% Medicare beneficiaries in higher income groups who received care from internists had undergone testing, which is nearly at the national screening target proposed for 2020. We observed the lowest rate, 34%, among disabled enrollees (50-54 years old) receiving care from FPs, which was 50% lower than the rate for this group of enrollees who had received care from internists. Only about half of eligible patients had received an FOBT kit, with proportionally fewer of FPs' patients receiving one. Less than a fifth of beneficiaries who received care from FPs received a screening colonoscopy recommendation, compared to about a third of those who received care from internists.

To our knowledge, no previous studies have simultaneously examined specialty of usual healthcare provider and patients' receipt of CRC screening in a nationally representative sample. That said, our findings with respect to specialty differences in screening rates are consistent with previous studies showing that among adult PCPs, internists have higher rates of providing preventive care services including cancer screening than FPs [16,17]. A study using Medicare claims data found that patients receiving care from FPs/GPs were less likely to undergo CRC screening compared to patients of internists [18]. Our study used data from in-person interviews with patients, supplemented with insurance claims, and included analyses on multiple CRC screening outcomes at both the patient and provider levels. This provides a more detailed analysis of patient characteristics, patient knowledge of screening services and use of screening tests based on the primary care specialist seen.

The most plausible reason for PCP specialty differences in screening is that family practitioners are less aggressive about offering CRC testing to their patients. Patients receiving care from FPs were equally knowledgeable about CRC screening and Medicare's benefit for screening, as those receiving care from internists. In fact, compared to internists, a higher percentage of patients receiving care from FPs had heard of colonoscopy, and yet screening-eligible patients of FPs were less likely to have been offered a home FOBT kit or a recommendation for colonoscopy. Our findings suggest that once offered screening, the rates of test completion were similar for both groups of patients. These findings suggest that the lower rates of CRC screening are due, in part, to potentially remediable healthcare provider variations in screening practices and not solely from patient-related factors or systematic refusal of testing by patients. Our results show that eliminating PCP variations in CRC practices has the potential to substantially increase utilization of CRC screening among a diverse group of screening-eligible adults.

There are other possible explanations for the PCP specialty variations found in this study. Some previous studies suggest that FPs may be more likely to provide safety-net care in non-academic and rural settings [17,18]. This could have a significant impact on recommendation for colonoscopy as FPs may have less access to subspecialist referral networks necessary for colonoscopy. Further, FPs provide a wider scope of services [17], which may lead to more competing and disparate demands in their practices than for internists [13]. However, in this study, the differences persisted even after controlling for patient factors, suggesting that differences in use of screening tests cannot be attributed to differences in the complexity of patients' medical care needs alone.

Organization and style of physician practices, such as tracking systems for monitoring delivery of CRC screening services, may contribute to the differences observed in this study [17,18]. Such practice-related barriers to providing CRC screening may present greater hurdles for FPs than internists, particularly in rural and underserved areas. Tailored practice-based interventions [25] including reminder systems [26], clinical outreach, programs to monitor disparate care in practices, and the use of preventive medicine specialists, supported by information technology solutions and incentive programs, may increase screening within primary care offices. Mitigating such hurdles has the potential to increase recommendations of CRC screening in primary care practices.

### Limitations and strengths

Measures of CRC testing were based on self-report, which can be subject to considerable recall bias. Self-report may overestimate screening rates [27-29], or, on the other hand, may capture information not captured in claims, particularly for those with supplemental insurance, who may not have valid claims in Medicare databases [30]. Also, the use of colonoscopy or sigmoidoscopy was defined as being within a 5-year time period rather than the 10-year period recommended by some guidelines. The screening rates may have been higher if the exposure measurement considered a 10-year period prior to the interview date rather than the interval used for this report. However, screening colonoscopy was relatively uncommon in the early 2000s [20]. Thus, extending the window for ascertainment of colonoscopy is unlikely to have a substantive impact on the findings. Also, we were unable to differentiate screening from diagnostic exams. These limitations may result in misclassification of some of the outcomes studied. The misclassification was likely non-differential and thus would not have changed our findings. This study was based on data on Medicare beneficiaries in the United States and as such, may not be generalized to other populations or settings with different health care systems. However, the findings provide important lessons for evaluating and improving the delivery of cancer screening services in primary care for a broad range of settings.

## Conclusions

The use of CRC screening tests was lower than the national goal despite high levels of CRC screening awareness irrespective of whether patients received care from internists or family physicians. Our results suggest that potentially remediable variations in practice style or practice environment contributed to the differences in screening rates according to PCP specialty. Our study shows the potential to greatly increase screening rates nationally through greater efforts to increase physicians' offering of CRC screening. However, additional studies, including mixed methods research that capture the experiences of patients in primary care who have not undergone testing, may provide data that can be used to develop interventions to improve delivery of CRC screening in primary care setting.

## Competing interests

The authors declare that they have no competing interests.

## Authors' contributions

AYH was actively involved in conceptualization, drafting and editing of the manuscript. AOL and ARBD participated actively in the conceptualization of the paper, analyses plan and provided critical reviews on the manuscripts. JT and BB provided interpretation of the data and critical review of the manuscript for important intellectual content. KLP participated in developing the analyses plan and critical reviews of the manuscript. CAD was responsible for conception, analysis and interpretation of the findings; and had full access to the data. All authors read and approved the final manuscript.

## Pre-publication history

The pre-publication history for this paper can be accessed here:

http://www.biomedcentral.com/1471-230X/12/23/prepub
